# Cross-sectional serosurvey of *Leptospira* species among slaughter pigs, goats, and sheep in Uganda

**DOI:** 10.1371/journal.pntd.0012055

**Published:** 2024-03-15

**Authors:** Lordrick Alinaitwe, Christopher Joshua Aturinda, Ashiraf Lubega, Velma Kivali, James Bugeza, Martin Wainaina, Martin H. Richter, Jolly Justine Hoona, Kristina Roesel, Anne Mayer-Scholl, Elizabeth Anne Jessie Cook, Clovice Kankya, Salome Dürr

**Affiliations:** 1 Human and Animal Health Program, International Livestock Research Institute, Nairobi, Kenya; 2 Veterinary Public Health Institute, University of Bern, Bern, Switzerland; 3 Graduate School for Cellular and Biomedical Sciences, University of Bern, Bern, Switzerland; 4 College of Veterinary Medicine, Animal Resources and Biosecurity (COVAB), Makerere University, Kampala, Uganda; 5 Vaccinology Research Program, National Livestock Resources Research Institute, Kampala, Uganda; 6 Department of Biological Safety, Federal Institute for Risk Assessment, Berlin, Germany; 7 Department of Animal Production, Ministry of Agriculture, Animal Industry and Fisheries (MAAIF), Entebbe, Uganda; Universidade Federal de Pelotas, BRAZIL

## Abstract

**Introduction:**

*Leptospira* are a group of bacteria, including pathogenic types that cause leptospirosis. In Uganda, *Leptospira* exposure has been reported in humans, with domesticated animals being speculated as the source. However, comparable evidence of *Leptospira* prevalence and circulating serovars/serogroups in animals is only documented for cattle, and dogs. Our study determined *Leptospira* seroprevalence, associated risk factors and serogroups circulating among slaughtered pigs, goats, and sheep in Uganda.

**Methods:**

During an 11-month cross-sectional survey in selected slaughter facilities in three regions of Uganda, we collected blood from 926 pigs, 347 goats, and 116 sheep. The age, sex, breed, and origin of each sampled animal were noted. The samples were tested for anti-*Leptospira* antibodies using the microscopic agglutination test, based on a panel of 12 serovars belonging to 12 serogroups.

**Results:**

*Leptospira* seroprevalence was 26.67% (247/926, 95%CI 23.92–29.61) among pigs, and 21.81% (101/463, 95%CI 18.29–25.80) in goats and sheep (small ruminants). *L*. *interrogans* Australis and *L*. *kirschneri* Grippotyphosa were the commonest serovars among pigs, as was *L*. *borgpetersenii* Tarassovi in small ruminants. Pigs sourced from the Eastern (Odds Ratio [OR] = 2.82, 95%CI 1.84–4.30) and Northern (OR = 3.56, 95%CI 2.52–5.02) regions were more likely to be seropositive, compared to those from the Central region. For small ruminants, being female (OR 2.74, 95% CI 1.69–4.57) and adult (OR 4.47, 95% CI 1.57–18.80) was significantly more associated with *Leptospira* seropositivity.

Conclusion/significance: Detection of a moderate seroprevalence, and several *Leptospira* serogroups among pigs, sheep, and goats from all regions of Uganda, supports existing reports in cattle and dogs, and implies widespread *Leptospira* exposure in domestic animals in Uganda. These findings may inform future programs for the control of leptospirosis in livestock in Uganda.

## Author summary

*Leptospira* are a group of bacteria, including pathogenic types that cause a zoonotic disease called leptospirosis. In Uganda, domesticated animals are implied as the source of human *Leptospira* exposures, yet comparable animal data is only available for cattle and dogs. Knowing that control of leptospirosis requires an understanding of infection sources and factors influencing transmission, our study aimed at determining *Leptospira* seroprevalence, associated risk factors, and serogroups circulating among the other domesticated animals in Uganda.

We detected a moderate seroprevalence, and several *Leptospira* serogroups among pigs and small ruminants from all regions of Uganda. We determined that pigs sourced from the Eastern and Northern regions were significantly more seropositive, and that being female, or adult (≥7 months old) was a risk factor for seropositivity among small ruminants. These results support existing reports in cattle and dogs and imply widespread *Leptospira* exposure among livestock in Uganda. The findings may inform future programs for the control of leptospirosis in livestock in Uganda.

## Introduction

*Leptospira* are spirochete bacteria, including pathogenic species that cause leptospirosis, which is an endemic disease in subtropical and tropical countries. To date, about 69 *Leptospira* species, and more than 300 serovars are known [1–3]. Certain serovars have been reported as regionally endemic and increasingly host-adapted serovars in several animal species, especially rodents [4,5]. For example, the brown Norwegian rat (*Rattus norvegicus*) is an important global host for serovar Icterohaemorrhagiae, pigs for Bratislava and cattle for Hardjo [4]. Animal hosts to which serovars have successfully adapted remain asymptomatic but capable of shedding leptospires in urine for prolonged periods, consequently contaminating water, and soil [4,5]. Transmission to humans and domestic animals usually occurs either through direct contact of mucosae with *Leptospira*-contaminated urine or indirectly via water and soil [4,5].

The global annual incidence of human leptospirosis is estimated at one million cases, resulting in approximately 58,900 deaths [6]. For East Sub-Saharan Africa, annual morbidity of 91,100 cases per 100,000 population has been reported [6]. In Uganda, *Leptospira* exposure has been shown in febrile patients from geographically distinct areas [7–10]. Domestic animals, particularly cattle and pigs are speculated as the source of these human *Leptospira* exposures [7,8,10], despite evidence of exposure in other main reservoirs such as wildlife [11]. This is possible since human-wildlife interactions are less common than interactions with domestic animals. In one study, *Leptospira* seroprevalence of 35% was reported, with patients who had been involved in skinning of cattle having 12 times higher odds of being seropositive [8]. *L*. *borgpetersenii* Nigeria from serogroup Pyrogenes was the most prevalent serovar in that study. Follow-up studies reported *Leptospira* seroprevalence of 19.3% among cattle on farms in the Northern and Eastern districts of Uganda [12], and 27.8% in cattle from a geographically wider survey in slaughter facilities [13]. However, serovar Nigeria was not as highly prevalent in cattle as was reported in humans, suggesting there may be other animal sources. *Leptospira* exposure had also been reported in dogs in Uganda, and to similar serogroups as reported in cattle [14]. Although this could mean endemicity and widespread exposure to leptospires among domestic animals in Uganda, comparable reports of *Leptospira* serovars or serogroups circulating in some domestic animal hosts are missing.

The epidemiology of leptospirosis is dynamic, even in endemic setups [15]. Thus, control and prevention strategies should be based on an updated understanding of the infection sources, infecting *Leptospira* types or serogroups and factors associated with transmission. This information can be derived from disease surveillance activities in targeted areas and populations. In this regard, slaughter facilities permit convenient sampling from large livestock populations in a wide geographical reach [16]. In the current study, we used slaughter facilities across three regions of Uganda, to determine the extent of *Leptospira* exposures, associated risk factors and circulating serogroups among pigs, goats, and sheep.

## Materials and methods

### Ethics statement

Study procedures were approved by the Institutional Animal Care and Use Committee of the International Livestock Research Institute (Approval Number ILRI-IACUC2022-17), the School of Biosecurity, Biotechnical and Laboratory Sciences, College of Veterinary Medicine, Animal Resources and Biosecurity (COVAB), Makerere University (Approval number SBLS/HDRC/20/012) and the Uganda National Council for Science and Technology (Approval Number HS1563ES). Written consent was obtained from the management of the slaughter facilities ahead of the study.

### Description of study area

**Fig 1:**
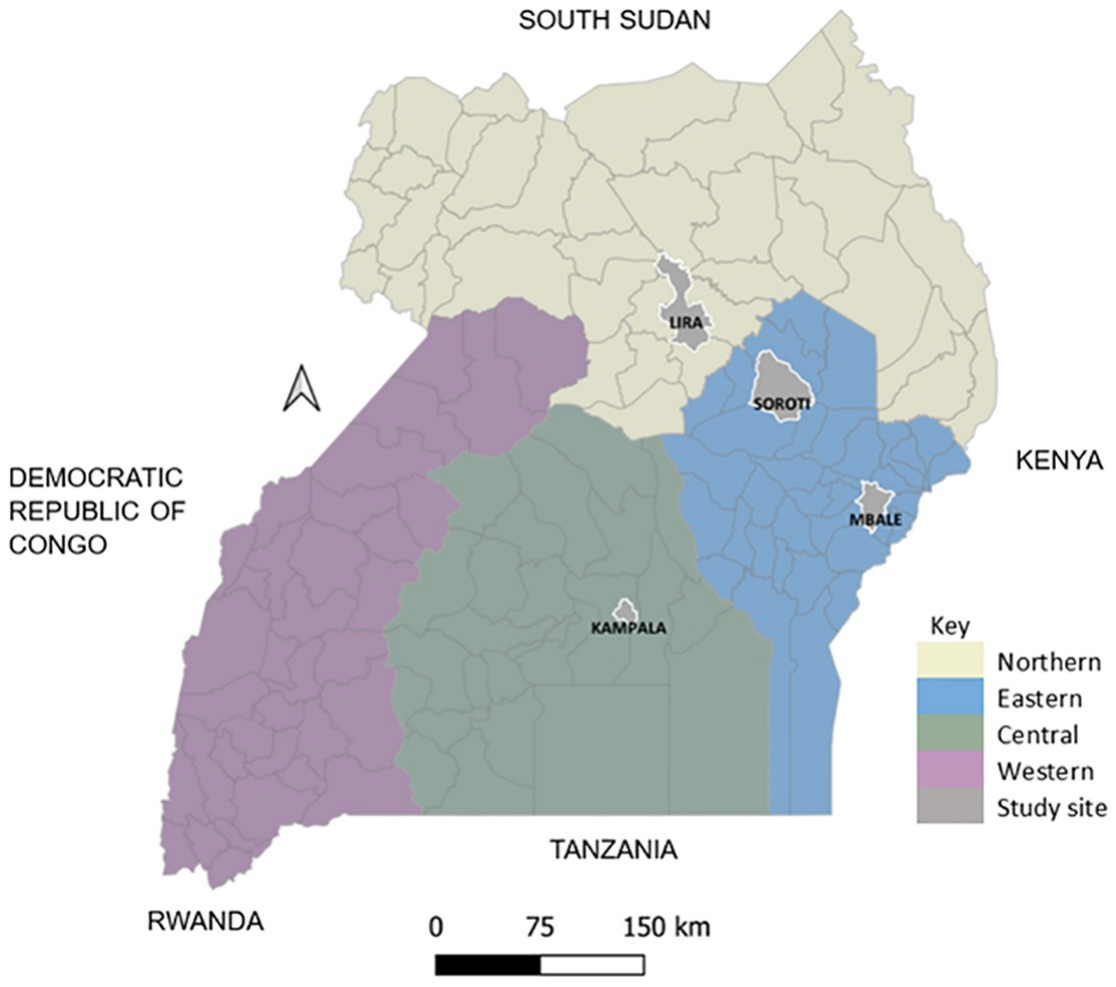
Map of Uganda showing the regions, districts, and sites selected for the cross-sectional serosurvey of *Leptospira* in slaughtered pigs and small ruminants. *Source*: *Map drawn in Open QGIS version 3*.*10*, *with a base layer of Uganda districts downloaded from the data portal of the United Nations High Commissioner for Refugees (*Document - Uganda Districts Shapefiles 2020 (unhcr.org).

Uganda is a land-locked country located in East Africa, with a land area of 241,551 km^2^ and a warm tropical climate [17]. The country is divided into four geographical regions (North, East, Central and West), and 135 districts as shown in Fig 1. The population of Uganda was estimated at 40.3 million people by mid-2019 and indicated to rapidly grow. Most of the people are involved in some form of crop or livestock farming, especially in the rural areas [17]. There are about 16.4 million goats, 14.6 million cattle, 4.6 million sheep and 4.2 million pigs in Uganda, making these the country’s most kept livestock species, after poultry. A large proportion of pigs are kept in the Central and Western regions [17]. The majority of cattle, sheep and goats are kept in a pastoralist-dominated continuous strip of land stretching from the Southwestern to the Northeastern part of the country, commonly known as the cattle corridor [18]. This corridor is target for local livestock trade, and has been shown to dominate supply of cattle to the largest slaughter facilities in Uganda [19].

### Study design

A cross-sectional study was conducted between December 2021 and October 2022, in selected livestock slaughter facilities across three of the four geographical regions of Uganda. In each region, one district that slaughtered the highest number of all livestock species, and in which the slaughter facilities supported our sampling strategy was selected as a study site. The initial list of target districts by region, livestock population and slaughter volume were generated with consultation from stakeholders in the Uganda Ministry of Agriculture, Animal Industry and Fisheries at project inception. Reconnaissance visits were then made to map slaughter facilities and assess their fitness to sampling strategy. The rationale was that the daily demand for slaughter animals in the large regional slaughter facilities is met by a supply from several districts within that and/or other regions, enabling a large geographically representative sample of livestock. The selected study sites were Lira in the Northern region, Kampala in the Central, Mbale (for goats and sheep) and Soroti (for pigs only) in the Eastern region (Fig 1). Two sites were recruited in the Eastern region since no single district slaughtered the highest number of all the livestock species. No site was recruited in the Western region following notification by key informants that a significant proportion of livestock slaughtered in Kampala (our study site in Central) came from the Western region, a fact also indicated by a previous study in slaughter cattle [19].

### Sample size estimation

Sample sizes were calculated in *epitools* epidemiological calculators [20], considering an imperfect microscopic agglutination test (MAT) with sensitivity and specificity of 55% and 97%, respectively, in an asymptomatic population [21], confidence level of 0.95 and precision of 0.05. The design seroprevalences for the sample size calculation were based on reports from countries neighboring Uganda due to missing local reports. The sample size estimates were: 915 pigs, based on a seroprevalence of 32.9% as reported in Kenyan slaughter pigs [22], and 403 for goats and sheep altogether, based on 8.5% seroprevalence reported in Tanzania [23]. Goats and sheep are considered a single population because we assume similar exposure risk, since they are always grazed and housed together.

### Sampling strategy and sample collection

In Lira and Mbale, small ruminants (sheep and goat) were sampled from two slaughter facilities with the highest daily slaughter numbers. There were two equally large ruminant slaughter facilities in Kampala, but for convenience we sampled from one. Samples from small ruminants were collected for 30 days at each site, skipping a day in between collections, to minimize over-representation of animals with the same population characteristics, for example coming from the same herd. In all sites, pigs were sampled from one facility, for 16 consecutive days, except in Soroti, where it lasted 10 days due to anticipated interference by Easter festivals. To compensate for the time difference, a second pig slaughter facility was enrolled. Consecutive sampling of pigs was considered because the daily slaughter stock turnover ranged between 80–100% in all sites at the time.

On each collection day, slaughtered animals were sampled opportunistically (next animal selected when sampling of the current one was completed). For each selected animal, blood from the cut neck vessels was collected into a sterile, single-use 50 or 120-mL container, and immediately transferred into an 8.5 mL serum separator tube (BD Vacutainer Ref 367958). The age (young, adult), sex (male, female), and breed (local, exotic, or cross) were noted. Age was based on empirical knowledge, with adult small ruminant being older than approximately seven months and pigs about six months or older. Assignment of breed was based on phenotypic characteristics of the animals. Information on the district of origin was often obtained from the traders or at times animal movement permits held at the slaughter facilities. Samples were transported daily in an ice-cooled box to the Central Diagnostic Laboratory at COVAB, Makerere University, Uganda. At the laboratory, blood was spun at 3,857 x g for 5 minutes, and serum separated into cryogenic tubes for storage at -20°C until tested.

### Serological testing

The MAT as recommended by the World Organization for Animal Health (WOAH) was used to determine presence of anti-*Leptospira* antibodies [24]. The MAT panel comprised of 12 serovars representing 12 serogroups (Table 1), and considered serogroups previously described as prevalent in Uganda [8,12,13], and those maintained in livestock elsewhere in East Africa [22,23,25]. All the serovars on the panel belong to pathogenic *Leptospira* species, which are known to infect humans and/or animals [1]. Strains of serovars used in the MAT were obtained from the Leptospirosis Reference Centre (WOAH Reference Laboratory for Leptospirosis), Amsterdam, the Netherlands. The strains were maintained at 29.5°C in commercial formulations of Ellinghausen and McCullough Johnson and Harris (EMJH) medium supplemented with commercial albumin, polysorbate 80 and additional growth factors (BD Difco *Leptospira* Enrichment, product 279510). Briefly, seven-day old live *Leptospira* cultures were used to screen the serum samples at an initial dilution of 1:50. Samples with a positive reaction were then titrated in a serial two-fold dilution until 1:12800, to determine the titre (reciprocal of the highest serum dilution at which ≥ 50% of the leptospires remained agglutinated). Sera with a titre ≥100 were considered seropositive. The reported titres here are consensus readings of two proficient observers and include the volume of the antigen culture.

**Table 1:** Strains of pathogenic *Leptospira* (*L*.) species used as live antigens in the MAT during the *Leptospira* serosurvey in pigs and small ruminant in Uganda.

Genomospecies	Serogroup	Serovar	Strain
*L*. *interrogans*	Australis	Australis	Ballico
	Icterohaemorrhagiae	Icterohaemorrhagiae	RGA
	Pomona	Pomona	Pomona
	Canicola	Canicola	Hond Utrecht IV
	Hebdomadis	Hebdomadis	Hebdomadis
*L*. *kirschneri*	Autumnalis	Butembo	Butembo
	Grippotyphosa	Grippotyphosa	Duyster
*L*. *borgpetersenii*	Pyrogenes	Nigeria	Vom
	Tarassovi	Tarassovi	Perepelitsin
	Sejroe	Sejroe	M84
	Ballum	Kenya	Njenga
*L*. *weilii*	Celledoni	Celledoni	Celledoni

### Data analysis

Data was entered in Microsoft Excel from Microsoft office 365 (Version 2306 Build 16.0.16529.20164), and analysed in R version 4.1.1 [26]. Seroprevalence by category of livestock (pigs or small ruminants) was calculated using the *epi*.*prev* function in the *EpiR* package. For each category of livestock, association between seropositivity and exposure variables (age, sex, breed, and region of origin) was analysed by entering all variables in a multivariable logistic regression model. A manual backward selection method was used to control for confounding variables, and only variables with a *P* value <0.05 were retained in the final model. To reduce the degrees of freedom, the seven exotic pigs were added to the same category as crossed breeds, since the two are genetically closer, compared to local breeds. The variable “region of origin” was generated by assigning the district from which the sampled animal was sourced to the respective geographical region of Uganda. The one pig from the Western region was excluded from the analysis. Model fitness was assessed using the Hosmer and Lemeshow goodness of fit test.

## Results

### Study population characteristics

Irrespective of the species, most of the animals sampled were adult (68.18%) and female (56.37%). Small ruminants were predominantly of breeds local to Uganda, while 66.41% of the pigs were of crossed breeds (Table 2). Facilities in Kampala slaughtered the largest number of animals, accounting for 56.66% (787/1389) of the total samples collected, despite ensuring similar sampling duration in the other study sites. A total of 615 pigs and 172 small ruminants were sampled from slaughter facilities in Kampala. There were 210 pigs and 172 small ruminants sampled from slaughter facilities in Lira, while the count in Mbale was 119 small ruminants, and in Soroti, 101 pigs. Small ruminants sampled in Kampala and Mbale were sourced from several regions of Uganda (S1 Fig). The district of origin for 29 animals (2.09% of the total sampled), could not be determined due to lack of access to accompanying documentation (Table 2).

**Table 2:** Composition by species, sex, age, breed and origin of the livestock sampled during the cross-sectional survey of *Leptospira* species in Uganda (N = 1389).

Variable	Level	Number of livestock species (%)			
		Pigs	Goats	Sheep	Total
Sex	Male	393 (42.44)	161 (46.40)	52 (44.83)	606 (43.63)
	Female	533 (57.56)	186 (53.60)	64 (55.17)	783 (56.37)
Age	Adult	542 (58.53)	301 (86.74)	104 (89.66)	947 (68.18)
	Young	384 (41.47)	46 (13.26)	12 (10.34)	442 (31.82)
Breed	Cross	615 (66.41)	61 (17.58)	2 (1.72)	678 (48.81)
	Exotic	7 (0.76)	0 (0)	0 (0)	7 (0.50)
	Local	304 (32.83)	286 (82.42)	114 (98.28)	704 (50.68)
Region of origin	Northern	210 (22.68)	203 (58.50)	72 (62.07)	485 (34.92)
	Eastern	119 (12.85)	39 (11.24)	5 (4.31)	163 (11.74)
	Western	1 (0.11)	65 (18.73)	25 (21.55)	91 (6.55)
	Central	586 (63.28)	28 (8.07)	7 (6.03)	621 (44.71)
	Undetermined	10 (1.08)	12 (3.46)	7 (6.03)	29 (2.09)
**Total**		**926 (100%)**	**347 (100%)**	**116 (100%)**	**1389 (100%)**

### *Leptospira* seroprevalence

**Fig 2:**
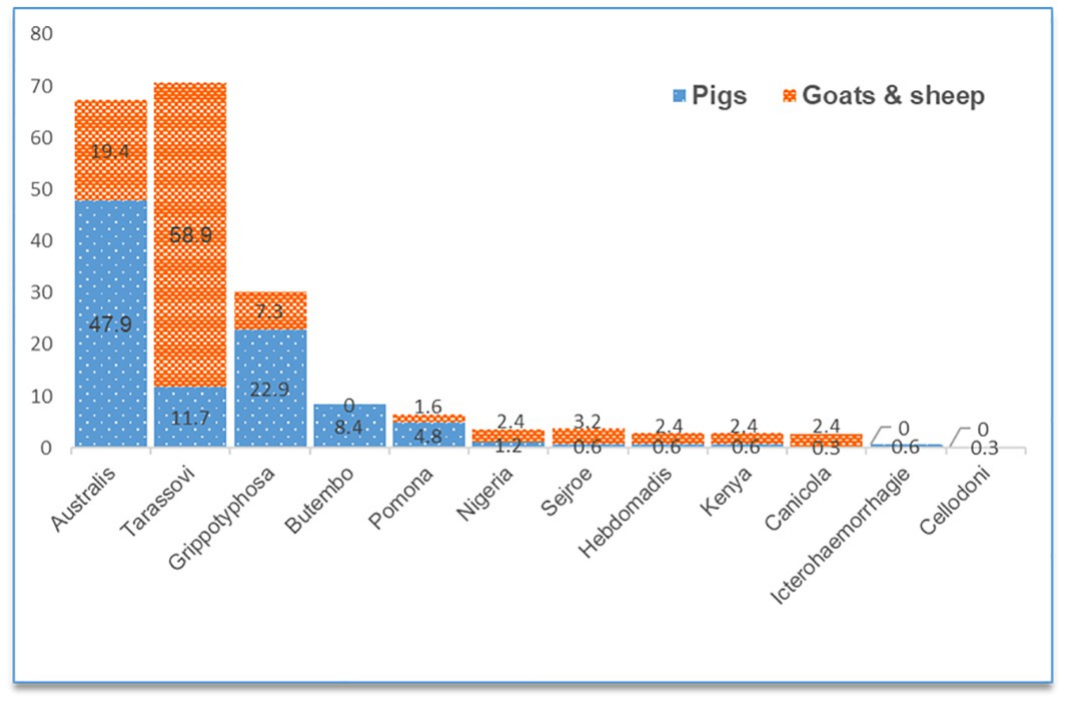
Proportion (%) of reactions to various *Leptospira* serovars among the seropositive pigs (n = 332), and small ruminants (n = 124) as determined by the microscopic agglutination test.

*Leptospira* seroprevalence as determined by MAT titre ≥100 was 26.67% (247/926, 95% confidence intervals [CI] = 23.92–29.61) among pigs, and 21.81% (101/463, 95% CI = 18.29–25.80) in small ruminants. Most of the seropositive pigs reacted to serovar *L*. *interrogans* Australis (47.9%) and *L*. *kirschneri* Grippotyphosa (22.9%), while small ruminants reacted more to *L*. *borgpetersenii* Tarassovi (58.9%) and *L*. *interrogans* Australis (19.4%), as shown in Fig 2. *Leptospira* seropositivity was highest in pigs that were sourced from the Northern region (44.29%), and in small ruminants from the Central region (31.43%). Adult and female livestock of all species were more seropositive compared to their young and male counterparts (Table 3). Seropositivity to multiple *Leptospira* serovars was detected in 7.02% (65/926) of the pigs and 3.89% (18/463) of the small ruminants. Most multiple reactions involved Australis with Grippotyphosa (35 counts), or Australis with Tarassovi (22 counts) (S1 Table). Up to 1.62% (15/926) of the pigs, and 2.59% (12/463) of the small ruminants sampled had high anti-*Leptospira* antibody titres (≥800), indicating probable recent infection (S2 and S3 Tables).

**Table 3:** Frequency of *Leptospira* seropositive samples by population characteristics of pigs (N = 926) and small ruminants (N = 463) sampled during a cross serosurvey in Ugandan slaughter facilities.

Variable	Level	Pigs		Small ruminants	
		Pos/n	% Positive	Pos/n	% Positive
Sex	Female	152/533	28.52	75/250	30.00
	Male	95/393	24.17	26/213	12.21
Age	Adult	163/542	30.07	98/405	24.20
	Young	84/384	21.88	3/58	5.17
Breed	Cross	163/615	26.50	13/63	20.63
	Exotic	1/7	14.29	0	0
	Local	83/304	27.30	88/400	22.00
Region of Origin	Northern	93/210	44.29	61/275	22.18
	Eastern	46/119	38.66	4/44	9.09
	Western	0/1	0	22/90	24.44
	Central	107/586	18.26	11/35	31.43
	Undetermined	1/10	10.00	3/19	15.79

Pos, the number of positive animals; n, the total number of animals in the variable level

### Risk factors for *Leptospira* seroprevalence

In the final regression model in pigs, *Leptospira* seropositivity was only significantly associated with region of origin (Table 4). Odds were 2.82 (95% CI 1.84–4.30) and 3.56 (95% CI 2.52–5.02) times higher for pigs sourced from the Eastern and Northern region, respectively, when compared to the Central region. In small ruminants, female (Odds Ratio (OR) 2.74, 95% CI 1.69–4.57) and adult (OR 4.47, 95% CI 1.57–18.80) small ruminants were significantly more seropositive (Table 4). There was no significant association between *Leptospira* seropositivity and breed or region of origin of small ruminants.

**Table 4:** Factors associated with exposure to leptospires among slaughter pigs and small ruminants in Uganda, as determined in the final multivariable logistic regression models.

Species	Category	Level	Odds ratio	95%CI	P–value
Small ruminants	Sex	Male	Ref		
		Female	2.74	1.69–4.57	<0.001
	Age	Young	Ref		
		Adult	4.47	1.57–18.80	0.014
*Hosmer and Lemeshow*: *X*^*2*^ = *0*.*018*, *p-value* = *0*.*894*, *number of bins (g) = 3*					
Pigs	Region of origin	Central	Ref		
		Eastern	2.82	1.84–4.30	<0.001
		Northern	3.56	2.52–5.02	<0.001
		Undetermined	0.50	0.03–2.69	0.510
*Hosmer and Lemeshow*: *X*^*2*^ *< 0*.*0001*, *p-value = 1*, *number of bins (g) = 3*					

Ref, the level in the category to which the others were compared; CI, confidence interval.

## Discussion

In this study, 26.67% of pigs and 21.81% of goats and sheep (small ruminants) sampled were seropositive for *Leptospira*, mostly of the Tarassovi, Australis and Grippotyphosa serogroups. Region of origin was a risk factor for *Leptospira* exposure in pigs while exposure in small ruminants was associated with age and sex. *Leptospira* seroprevalence reported in the current study may be higher than on-farm seroprevalence, especially for pigs, because of possible selection bias associated with sampling from slaughter animals. In endemic setups, farmers may cull animals based on disease-associated characteristics such as old age or poor reproductive performance [27]. Nevertheless, our study provides useful insights on *Leptospira* exposure in Ugandan livestock.

Similar seroprevalence levels and predominance of *Leptospira* serovars *L*. *interrogans* Australis and *L*. *borgpetersenii* Tarassovi was reported earlier in dogs (25.9%) and cattle (27.8%) from various locations of Uganda [13,14]. *Leptospira* infection of 10% has also been shown by PCR in kidney and reproductive tracts of slaughter pigs in Uganda, although the infecting serovars could not be concluded, making it difficult to compare these findings to our study [28]. Nonetheless, PCR based molecular assays permit more robust detection of ongoing *Leptospira* infection and downstream identification of serogroups/serovars that are local to the study area [29]. In this regard, molecular assays are superior to the MAT that relies on the representativeness of the selected reference serovars. Therefore, future studies in Uganda may test livestock by molecular assays to confirm if they are carriers of leptospires and detect locally circulating serovars.

The prevalence of Icterohaemorrhagiae, a rodent-associated serovar was rather low in the current study. This agrees with previous studies conducted in cattle and humans [8,12,13] and raises questions on the role of rodents in maintenance and transmission of *Leptospira* in Uganda. It is possible that rodents in Uganda reserve different serovars/serogroups from those used on the MAT panels or that the level of environmental contamination by rodents is generally too low to permit an indirect transmission. Future studies may consider determining *Leptospira* genome species reserved by rodents from several ecological sites and compare these to what is found in livestock and humans, to identify their potential role in *Leptospira* transmission pathways in Uganda.

Important from an animal productivity and public health perspective was the number of animals (n = 27, 1.94%) in the current study with high antibody titres (≥800) that may indicate recent infection. *Leptospira* infection in livestock results in production losses, from reduced milk yield in dairy herds, stunting in young animals, abortions, and sometimes deaths, depending on the infecting *Leptospira* species. Infected livestock may carry leptospires in their kidneys and shed the bacteria in urine, consequently increasing risk of transmission to other animals as well as humans [4,5]. The risk may be higher for individuals who are in frequent contact with livestock, especially slaughterhouse workers. *Leptospira* exposure among slaughterhouse workers has been reported in other East African countries [30,31], highlighting need for similar investigations in Uganda.

Finding the same serovars circulating in small ruminants and pigs could suggest interspecies transmission. Infection via contaminated environmental sources is a plausible explanation for this, given the farming practices. Small ruminants in Uganda are mainly kept by free-range during which infected ones could shed leptospires into water, soil, and vegetation. While the majority of pig farms in Uganda are semi-intensive, it is also common for subsistence farmers to free-range or tether pigs, particularly in the Northern and Eastern regions [32,33]. This may also be the reason for the significantly higher *Leptospira* seroprevalence in pigs from the Northern and Eastern regions, compared to the Central region. In some parts of Northern Uganda, free-ranging of pigs is more common in the rainy months of October-April [32], when the surface runoffs could facilitate survival and spread of leptospires in soil and water. The lack of data on pigs from the Western region may generate a sampling bias but is not expected to undermine the observed association between seropositivity and region of origin in the current study. The husbandry practices and breeds of pigs kept in the Western region are comparable to the Central region [34,35], making it reasonable to assume that the level of seropositivity is similar between the two regions.

The observed high *Leptospira* seropositivity in adult small ruminants could be because adult animals are more exposed to *Leptospira* reservoirs in their endemic environments for having lived longer than their young counterpart. However, sampling from slaughter populations like in our case may mean over-representation of adult animals, since selection of slaughter animals is mostly based on size or weight, which correlates with age. Finding a plausible explanation for the significantly high *Leptospira* seropositivity in female small ruminants in the current study is difficult, as the relationship between sex and immunity of animals to infectious diseases is inconsistently reported [36]. This association should also be cautiously extrapolated to the general population due to potential for over-representation of adult female animals. Female livestock sold for slaughter are often older than their male counterpart, since farmers selectively keep females born on farm for reproduction [37]. Additionally, selection of slaughter animals based on disease associated reproduction performance may be more common among adult females, increasing chances of *Leptospira* seropositive female animals being selected for slaughter. No wonder, studies conducted in slaughter facilities have consistently had larger proportions of female animals, and found high exposures among females, irrespective of the disease being investigated [22,28,38].

We found a higher *Leptospira* seroprevalence than was reported elsewhere in East Africa. For example; *Leptospira* seroprevalence of 8.47% (n = 248) was reported in goats and sheep of Tanzania, though a higher test cut-off titre (≥160) was used [23]. When the cut-off in our study is raised to ≥200, the prevalence would be about 14.25%. In Kenya, prevalence of 32.9% (cut-off titre ≥40) was shown in slaughter pigs, with *L*. *interrogans* serovar Lora (serogroup Australis) being highly prevalent [22]. At a cut-off ≥160, the authors reported seroprevalence of 6.8% (17/252) which is lower than 17.9 3% if we considered a cutoff of ≥200 in our study. A weighted seroprevalence of 15% was reported from a longitudinal study in small ruminants of Tana River County, Kenya, with similar circulating serogroups as found in our study [39]. This study did not test Tarassovi, the commonest serogroup in small ruminants in our study. Tanzania and Kenya are neighbors with Uganda, and the three countries share similar ecology [40]. This may mean that the same factors influence *Leptospira* transmission. Transboundary movement of livestock between these countries could also contribute to the observed similarity in circulating serovars [41].

Our study fills the knowledge gap on *Leptospira* exposure and circulating serogroups in pigs, goats, and sheep. This, together with previous reports in cattle and dogs [12–14], implies that leptospirosis is endemic in Uganda, and widespread in domestic animals. These findings may inform future programs for the control of leptospirosis in livestock in Uganda.

## Supporting information

S1 Table *Leptospira* serovars that are involved in multiple exposures. (DOCX)

S2 Table Prevalence and titres of serovar-specific anti-*Leptospira* antibodies in pigs. (DOCX)

S3 Table Prevalence and titres of serovar-specific anti-*Leptospira* antibodies in small ruminants. (DOCX)

S1 Fig Proportion of pigs and small ruminants sampled in each study site that were sourced from the four regions of Uganda. (TIFF)

## References

[1] Vincent AT, Schiettekatte O, Goarant C, Neela VK, Bernet E, Thibeaux R, et al. Revisiting the taxonomy and evolution of pathogenicity of the genus Leptospira through the prism of genomics. PLoS Negl Trop Dis. 2019;13: e0007270. doi: 10.1371/journal.pntd.0007270 31120895 PMC6532842

[2] Korba AA, Lounici H, Kainiu M, Vincent AT, Mariet J-F, Veyrier FJ, et al. Leptospira ainlahdjerensis sp. nov., Leptospira ainazelensis sp. nov., Leptospira abararensis sp. nov. and Leptospira chreensis sp. nov., four new species isolated from water sources in Algeria. Int J Syst Evol Microbiol. 2021;71: 5148. doi: 10.1099/ijsem.0.005148 34914572

[3] Fernandes LG V, Stone NE, Roe CC, Goris MGA, van der Linden H, Sahl JW, et al. Leptospira sanjuanensis sp. nov., a pathogenic species of the genus Leptospira isolated from soil in Puerto Rico. Int J Syst Evol Microbiol. 2022;72: 5560. 10.1099/ijsem.0.005560 36260655

[4] Ellis WA. Animal Leptospirosis. Leptospira and Leptospirosis. Springer; 2015. pp. 99–137. 10.1007/978-3-662-45059-8_6 25388134

[5] Bharti AR, Nally JE, Ricaldi JN, Matthias MA, Diaz MM, Lovett MA, et al. Leptospirosis: a zoonotic disease of global importance. Lancet Infect Dis. 2003;3: 757–771. doi: 10.1016/s1473-3099(03)00830-2 14652202

[6] Costa F, Hagan JE, Calcagno J, Kane M, Torgerson P, Martinez-Silveira MS, et al. Global morbidity and mortality of leptospirosis: a systematic review. PLoS Negl Trop Dis. 2015;9: e0003898. doi: 10.1371/journal.pntd.0003898 26379143 PMC4574773

[7] Schiff SJ, Kiwanuka J, Riggio G, Nguyen L, Mu K, Sproul E, et al. Separating Putative Pathogens from Background Contamination with Principal Orthogonal Decomposition: Evidence for Leptospira in the Ugandan Neonatal Septisome. Frontiers in Medicine. 2016. doi: 10.3389/fmed.2016.00022 27379237 PMC4904006

[8] Dreyfus A, Dyal JW, Pearson R, Kankya C, Kajura C, Alinaitwe L, et al. Leptospira Seroprevalence and Risk Factors in Health Centre Patients in Hoima District, Western Uganda. PLoS Negl Trop Dis. 2016;10: e0004858. doi: 10.1371/journal.pntd.0004858 27487398 PMC4972303

[9] Wambi R, Worodria W, Muleme J, Aggrey S, Mugisha L. Prevalence of leptospirosis among patients attending renal and general outpatient clinics in Mulago Hospital, Kampala, Uganda. Sci Rep. 2022;12: 1–7. 35589947 10.1038/s41598-022-12544-3 PMC9120167

[10] Kigozi BK, Kharod GA, Bukenya H, Shadomy S V, Haberling DL, Stoddard RA, et al. Investigating the etiology of acute febrile illness: a prospective clinic-based study in Uganda. BMC Infect Dis. 2023;23: 1–17. 37328808 10.1186/s12879-023-08335-4 PMC10276394

[11] Atherstone C, Picozzi K, Kalema-Zikusoka G. Seroprevalence of Leptospira hardjo in cattle and African buffalos in southwestern Uganda. Am J Trop Med Hyg. 2014;90: 288–290. doi: 10.4269/ajtmh.13-0466 24323512 PMC3919234

[12] Dreyfus A, Odoch T, Alinaitwe L, Rodriguez-Campos S, Tsegay A, Jaquier V, et al. Cross-Sectional serological survey for leptospira spp.In beef and dairy cattle in two districts in Uganda. Int J Environ Res Public Health. 2017;14. doi: 10.3390/ijerph14111421 29160792 PMC5708060

[13] Alinaitwe L, Kankya C, Namanya D, Pithua P, Dreyfus A. Leptospira Seroprevalence Among Ugandan Slaughter Cattle: Comparison of Sero-Status With Renal Leptospira Infection. Frontiers in Vet Sci. 2020. p. 106. doi: 10.3389/fvets.2020.00106 32185188 PMC7058543

[14] Millán J, Chirife AD, Kalema-Zikusoka G, Cabezón O, Muro J, Marco I, et al. Serosurvey of dogs for human, livestock, and wildlife pathogens, Uganda. Emerg Infect Dis. 2013;19: 680. doi: 10.3201/eid1904.121143 23750507 PMC3647413

[15] Thornley CN, Baker MG, Weinstein P, Maas EW. Changing epidemiology of human leptospirosis in New Zealand. Epidemiol Infect. 2002;128: 29–36. doi: 10.1017/s0950268801006392 11895088 PMC2869792

[16] García-Díez J, Saraiva S, Moura D, Grispoldi L, Cenci-Goga BT, Saraiva C. The Importance of the Slaughterhouse in Surveilling Animal and Public Health: A Systematic Review. Vet Sci. 2023;10: 167. doi: 10.3390/vetsci10020167 36851472 PMC9959654

[17] Uganda Bureau of Statistics. 2019 Statistical Abstract. 2019. https://www.ubbos.org/wp-content/uploads/publications/01_20202019_Statistical_Abstract_-Final.pdf

[18] Nansamba M, Sibiya J, Tumuhimbise R, Ocimati W, Kikulwe E, Karamura D, et al. Assessing drought effects on banana production and on-farm coping strategies by farmers—A study in the cattle corridor of Uganda. Clim Change. 2022;173: 21.

[19] Alinaitwe L, Kankya C, Allan KJ, Rodriguez-Campos S, Torgerson P, Dreyfus A. Bovine leptospirosis in abattoirs in Uganda: Molecular detection and risk of exposure among workers. Zoonoses Public Health. 2019;66. doi: 10.1111/zph.12616 31250522

[20] Sergeant ESG. Epitools epidemiological calculators. 2018.

[21] Schlichting D, Nöckler K, Bahn P, Luge E, Greiner M, Müller-Graf C, et al. Estimation of the sensitivity and specificity of a Leptospira spp. in-house ELISA through Bayesian modelling. Int J Med Microbiol. 2015;305: 756–761. doi: 10.1016/j.ijmm.2015.08.029 26358915

[22] Ngugi JN, Fèvre EM, Mgode GF, Obonyo M, Mhamphi GG, Otieno CA, et al. Seroprevalence and associated risk factors of leptospirosis in slaughter pigs; a neglected public health risk, western Kenya. BMC Vet Res. 2019;15: 403. doi: 10.1186/s12917-019-2159-3 31703588 PMC6842184

[23] Assenga JA, Matemba LE, Muller SK, Mhamphi GG, Kazwala RR. Predominant leptospiral serogroups circulating among humans, livestock and wildlife in Katavi-Rukwa ecosystem, Tanzania. PLoS Negl Trop Dis. 2015;9: e0003607. doi: 10.1371/journal.pntd.0003607 25806825 PMC4373666

[24] WAHO. Manual of Diagnostic Tests and Vaccines for Terrestrial Animals, twelfth edition 2023. 2023. https://www.woah.org/fileadmin/Home/eng/Health_standards/tahm/3.01.12_LEPTO.pdf

[25] Schoonman L, Swai ES. Herd- and animal-level risk factors for bovine leptospirosis in Tanga region of Tanzania. 2010; 1565–1572. doi: 10.1007/s11250-010-9607-1 20517645

[26] R Core Team A, Team RC. R: A language and environment for statistical computing. R Foundation for Statistical Computing, Vienna, Austria. 2012. 2022.

[27] McKenna SLB, Keefe GP, Barkema HW, McClure J, VanLeeuwen JA, Hanna P, et al. Cow-level prevalence of paratuberculosis in culled dairy cows in Atlantic Canada and Maine. J Dairy Sci. 2004;87: 3770–3777. doi: 10.3168/jds.S0022-0302(04)73515-8 15483160

[28] Atherstone C, Mgode GF, Dhand NK, Alonso S, Grace D, Ward MP, et al. Selected endemic zoonoses in pigs presenting for slaughter in Kampala, Uganda. Am J Trop Med Hyg. 2020;103: 2552. doi: 10.4269/ajtmh.20-0033 33069266 PMC7695076

[29] Cerqueira GM, Picardeau M. A century of Leptospira strain typing. Infect Genet Evol. 2009;9: 760–768. doi: 10.1016/j.meegid.2009.06.009 19540362

[30] Cook EAJ, de Glanville WA, Thomas LF, Kariuki S, de Clare Bronsvoort BM, Fèvre EM. Risk factors for leptospirosis seropositivity in slaughterhouse workers in western Kenya. Occup Environ Med. 2017;74: 357–365. doi: 10.1136/oemed-2016-103895 27913579 PMC5520261

[31] Mirambo MM, Mgode GF, Malima ZO, John M, Mngumi B, Mhamphi GG, et al. Seropositivity of Brucella spp. and Leptospira spp. antibodies among abattoir workers and meat vendors in the city of Mwanza, Tanzania: A call for one health approach control strategies. 2018; 39–52. 10.1371/journal.pntd.0006600 PMC6034905 29939991

[32] Chenais E, Boqvist S, Sternberg-Lewerin S, Emanuelson U, Ouma E, Dione M, et al. Knowledge, attitudes and practices related to African swine fever within smallholder pig production in northern Uganda. Transbound Emerg Dis. 2017;64: 101–115. 25876769 10.1111/tbed.12347

[33] Nantima N, Ocaido M, Ouma E, Davies J, Dione M, Okoth E, et al. Risk factors associated with occurrence of African swine fever outbreaks in smallholder pig farms in four districts along the Uganda-Kenya border. Trop Anim Health Prod. 2015;47: 589–595. doi: 10.1007/s11250-015-0768-9 25616986

[34] Kampire J, Rugunda KG, Kiwanuka GN. Distribution and relative abundance of pig breeds in South-Western Agro-ecological Zone, Uganda: Status of locally adapted pigs. 2023.

[35] Muhanguzi D, Lutwama V, Mwiine FN. Factors that influence pig production in Central Uganda-Case study of Nangabo Sub-County, Wakiso district. 2012.

[36] Kelly CD, Stoehr AM, Nunn C, Smyth KN, Prokop ZM. Sexual dimorphism in immunity across animals: a meta-analysis. Ecol Lett. 2018;21: 1885–1894. doi: 10.1111/ele.13164 30288910

[37] Nsoso SJ, Podisi B, Otsogile E, Mokhutshwane BS, Ahmadu B. Phenotypic Characterization of Indigenous Tswana Goats and Sheep Breeds in Botswana: Continuous Traits. Trop Anim Health Prod. 2004;36: 789–800. doi: 10.1023/b:trop.0000045979.52357.61 15643814

[38] Bugeza JK, Roesel K, Moriyón I, Mugizi DR, Alinaitwe L, Kivali V, et al. Sero-prevalence and factors associated with anti-Brucella antibodies in slaughter livestock in Uganda. Front Epidemiol. 2023;3: 1213592. doi: 10.3389/fepid.2023.1213592 38455915 PMC10910896

[39] Wainaina M, Lindahl JF, Dohoo I, Mayer-Scholl A, Roesel K, Mbotha D, et al. Longitudinal Study of Selected Bacterial Zoonoses in Small Ruminants in Tana River County, Kenya. Microorganisms. 2022;10: 1546. doi: 10.3390/microorganisms10081546 36013964 PMC9414833

[40] Wei F, Wang S, Fu B, Liu Y. Representation of biodiversity and ecosystem services in East Africa’s protected area network. Ambio. 2020;49: 245–257. doi: 10.1007/s13280-019-01155-4 30852776 PMC6888792

[41] Zaal F, Siloma MO, Andiema R, Kotomei A. The geography of integration: cross-border livestock trade in East Africa. Pastor Livest Mark East Africa Res policy challenges. 2006; 145–168.

