# Supplementary material for: Cross-sectional serosurvey of Leptospira species among slaughter pigs, goats, and sheep in Uganda
Source: PLoS Negl Trop Dis. 2024 Mar 15;18(3):e0012055. doi: 10.1371/journal.pntd.0012055 (PMC10971767; doi:10.1371/journal.pntd.0012055)
Supplement: S1 Table — (DOCX) [file pntd.0012055.s001.docx]

S1 Table. *Leptospira* serovars that were involved in multiple exposures among the seropositive pigs and small ruminants sampled during a cross-sectional survey in Uganda.

| Pigs | | | | | | | | | | | |
| --- | --- | --- | --- | --- | --- | --- | --- | --- | --- | --- | --- |
|  | Icter | Pom | But | Grip | Heb | Ken | Nig | Cell | Aust | Tar | Can |
| Sej | 0 | 0 | 0 | 2 | 0 | 0 | 0 | 1 | 1 | 0 | 0 |
| Icter | Null | 0 | 0 | 0 | 0 | 0 | 0 | 0 | 1 | 0 | 0 |
| Pom | 0 | Null | 2 | 2 | 0 | 0 | 0 | 0 | 10 | 4 | 0 |
| But | 0 | 0 | Null | 8 | 0 | 0 | 0 | 0 | 11 | 6 | 0 |
| Grip | 0 | 0 | 0 | Null | 0 | 1 | 1 | 1 | 34 | 6 | 0 |
| Heb | 0 | 0 | 0 | 0 | Null | 0 | 0 | 0 | 0 | 0 | 0 |
| Ken | 0 | 0 | 0 | 0 | 0 | Null | 0 | 0 | 1 | 0 | 0 |
| Nig | 0 | 0 | 0 | 0 | 0 | 0 | Null | 0 | 0 | 1 | 0 |
| Cell | 0 | 0 | 0 | 0 | 0 | 0 | 0 | Null | 1 | 0 | 0 |
| Aus | 0 | 0 | 0 | 0 | 0 | 0 | 0 | 0 | Null | 14 | 0 |
| Tar | 0 | 0 | 0 | 0 | 0 | 0 | 0 | 0 | 0 | Null | 1 |
| Exposure to over two serovars | But-Aus-Tar (3), But-Grip-Aus (1), But-Grip-Aus-Tar (2), Grip-Aus-Tar (3), Grip-Ken-Aus (1), Grip-Nig-Tar (1), Pom-Aus-Tar (2), Pom-But-Grip-Aus (1), Pom-But-Tar (1), Sej-Grip-Can-Aus (1) | | | | | | | | | | |
| Small ruminants | | | | | | | | | | | |
|  | Icter | Pom | But | Grip | Heb | Ken | Nig | Cell | Aust | Tar | Can |
| Sej | 0 | 0 | 0 | 0 | 1 | 0 | 0 | 0 | 2 | 3 | 0 |
| Icter | Null | 0 | 0 | 0 | 0 | 0 | 0 | 0 | 0 | 0 | 0 |
| Pom | 0 | Null | 0 | 0 | 0 | 0 | 0 | 0 | 2 | 0 | 0 |
| But | 0 | 0 | Null | 0 | 0 | 0 | 0 | 0 | 0 | 0 | 0 |
| Grip | 0 | 0 | 0 | Null | 0 | 0 | 0 | 0 | 1 | 3 | 0 |
| Heb | 0 | 0 | 0 | 0 | Null | 0 | 0 | 0 | 1 | 0 | 0 |
| Ken | 0 | 0 | 0 | 0 | 0 | Null | 1 | 0 | 0 | 3 | 0 |
| Nig | 0 | 0 | 0 | 0 | 0 | 0 | Null | 0 | 0 | 1 | 0 |
| Cell | 0 | 0 | 0 | 0 | 0 | 0 | 0 | Null | 0 | 0 | 0 |
| Aus | 0 | 0 | 0 | 0 | 0 | 0 | 0 | 0 | Null | 8 | 0 |
| Tar | 0 | 0 | 0 | 0 | 0 | 0 | 0 | 0 | 0 | Null | 0 |
| Exposure to over two serovars | Aus-Tar-Can (1), Grip-Aus-Tar (1), Ken-Nig-Tar (1), Sej-Aus-Tar (2) | | | | | | | | | | |

*Sej-Sejroe, Icter-Incterohaemorrhagiae, Pom-Pomona, But-Butembo,Grip-Grippotyphosa, Heb-Hebdomadis, Ken-Kenya, Nig-Nigeria, Cell-Celledoni, Aus-Australis,Tar-Tarassovi, Can-Canicola*
