# Supplementary material for: Cross-sectional serosurvey of Leptospira species among slaughter pigs, goats, and sheep in Uganda
Source: PLoS Negl Trop Dis. 2024 Mar 15;18(3):e0012055. doi: 10.1371/journal.pntd.0012055 (PMC10971767; doi:10.1371/journal.pntd.0012055)
Supplement: S2 Table — (DOCX) [file pntd.0012055.s002.docx]

S2 Table. Prevalence and titres of serovar-specific anti-*Leptospira* antibodies measured by the microscopic agglutination test among slaughter pigs in Uganda (N = 926)

| **Serovar** | **100** | **200** | **400** | **800** | **1600** | **3200** | **Npos** | **%Positive**  **(CI)** |
| --- | --- | --- | --- | --- | --- | --- | --- | --- |
| Australis | 92 | 44 | 15 | 5 | 2 | 1 | 159 | 17.17  (14.81-19.74) |
| Grippotyphosa | 24 | 35 | 16 | 1 | 0 | 0 | 76 | 8.21  (6.54-10.17) |
| Tarassovi | 18 | 7 | 11 | 3 | 0 | 0 | 39 | 4.21  (3.03-5.68) |
| Butembo | 14 | 12 | 1 | 1 | 0 | 0 | 28 | 3.02  (2.06-4.33) |
| Pomona | 5 | 5 | 4 | 1 | 1 | 0 | 16 | 1.73  (1.03-2.77) |
| Nigeria | 4 | 0 | 0 | 0 | 0 | 0 | 4 | 0.43  (0. 14-1.09) |
| Sejroe | 2 | 0 | 0 | 0 | 0 | 0 | 2 | 0.22  (0.04-0.77) |
| Hebdomadis | 2 | 0 | 0 | 0 | 0 | 0 | 2 | 0.22  (0.04-0.77) |
| Kenya | 2 | 0 | 0 | 0 | 0 | 0 | 2 | 0.22  (0.04-0.77) |
| Icterohaemorrhagiae | 1 | 1 | 0 | 0 | 0 | 0 | 2 | 0.22  (0.04-0.77) |
| Canicola | 1 | 0 | 0 | 0 | 0 | 0 | 1 | 0.11  (0.0-0.60) |
| Celledoni | 1 | 0 | 0 | 0 | 0 | 0 | 1 | 0.11  (0.0-0.60) |
| Npos* titre | 166 | 104 | 47 | 11 | 3 | 1 | 332 |  |

*Npos* titre, the number of pigs with the various levels of antibodies against the respective Leptospira serovars/serogroups tested; CI, confidence interval*
