# Supplementary material for: Cross-sectional serosurvey of Leptospira species among slaughter pigs, goats, and sheep in Uganda
Source: PLoS Negl Trop Dis. 2024 Mar 15;18(3):e0012055. doi: 10.1371/journal.pntd.0012055 (PMC10971767; doi:10.1371/journal.pntd.0012055)
Supplement: S3 Table — (DOCX) [file pntd.0012055.s003.docx]

S3 Table. Prevalence and titres of serovar-specific anti-*Leptospira* antibodies measured by the microscopic agglutination test among slaughter goats and sheep in Uganda (N = 463)

| **Serovar** | **100** | **200** | **400** | **800** | **1600** | **3200** | **Npos** | **%Pos**  **(95% CI)** |
| --- | --- | --- | --- | --- | --- | --- | --- | --- |
| Tarassovi | 34 | 19 | 11 | 6 | 2 | 1 | 73 | 15.77  (12.66-19.38) |
| Australis | 5 | 10 | 6 | 1 | 2 | 0 | 24 | 5.18  (3.36-7.58) |
| Grippotyphosa | 8 | 1 | 0 | 0 | 0 | 0 | 9 | 1.94  (0.96-3.59) |
| Sejroe | 3 | 1 | 0 | 0 | 0 | 0 | 4 | 0.86  (0.29-2.18) |
| Nigeria | 2 | 1 | 0 | 0 | 0 | 0 | 3 | 0.65  (0.17-1.85) |
| Hebdomadis | 2 | 1 | 0 | 0 | 0 | 0 | 3 | 0.65  (0.17-1.85) |
| Kenya | 1 | 2 | 0 | 0 | 0 | 0 | 3 | 0.65  (0.17-1.85) |
| Canicola | 2 | 1 | 0 | 0 | 0 | 0 | 3 | 0.65  (0.17-1.85) |
| Pomona | 1 | 1 | 0 | 0 | 0 | 0 | 2 | 0.43  (0.07-1.53) |
| Butembo | 0 | 0 | 0 | 0 | 0 | 0 | 0 | 0  (0-0.77) |
| Icterohaemorrhagiae | 0 | 0 | 0 | 0 | 0 | 0 | 0 | 0  (0-0.77) |
| Celledoni | 0 | 0 | 0 | 0 | 0 | 0 | 0 | 0  (0-0.77) |
| Npos* titer | 58 | 37 | 17 | 7 | 4 | 1 | 124 |  |

*Npos* titre, the number of goats and sheep with the various levels of antibodies against the respective Leptospira serovars/serogroups tested; CI, confidence interval*
