# Supplementary figures and images for: Cross-sectional serosurvey of Leptospira species among slaughter pigs, goats, and sheep in Uganda
Source: PLoS Negl Trop Dis. 2024 Mar 15;18(3):e0012055. doi: 10.1371/journal.pntd.0012055 (PMC10971767; doi:10.1371/journal.pntd.0012055)

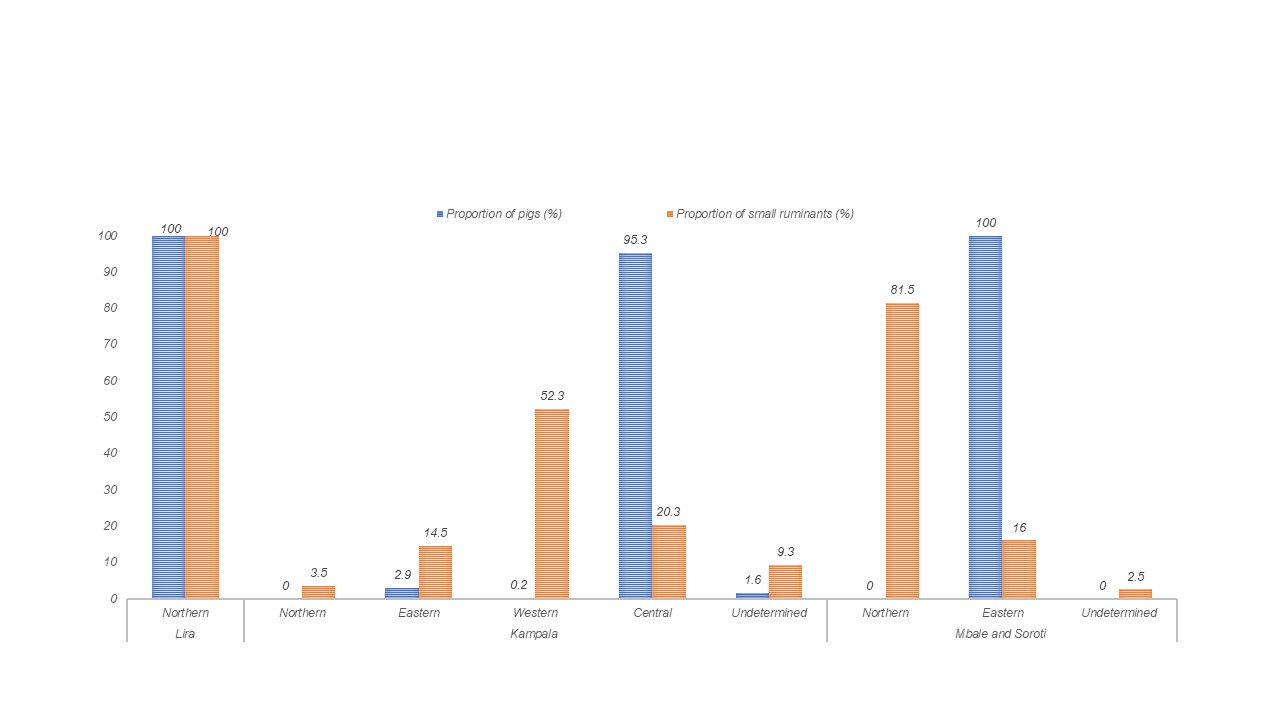

Supplement: S1 Fig — (TIFF) [file pntd.0012055.s004.tiff]
